# Compact solid-state optical phased array beam scanners based on polymeric photonic integrated circuits

**DOI:** 10.1038/s41598-021-90120-x

**Published:** 2021-05-19

**Authors:** Sung-Moon Kim, Eun-Su Lee, Kwon-Wook Chun, Jinung Jin, Min-Cheol Oh

**Affiliations:** Department of Electronics Engineering, Pusan National University, Pusan (Busan), 46241 Republic of Korea

**Keywords:** Electrical and electronic engineering, Integrated optics, Optical sensors, Optoelectronic devices and components

## Abstract

Optical phased array (OPA) devices are being actively investigated to develop compact solid-state beam scanners, which are essential in fields such as LiDAR, free-space optical links, biophotonics, etc. Based on the unique nature of perfluorinated polymers, we propose a polymer waveguide OPA with the advantages of low driving power and high optical throughput. Unlike silicon photonic OPAs, the polymer OPAs enable sustainable phase distribution control during beam scanning, which reduces the burden of beamforming. Moreover, by incorporating a tunable wavelength laser comprising a polymer waveguide Bragg reflector, two-dimensional beam scanning is demonstrated, which facilitates the development of laser-integrated polymeric OPA beam scanners.

## Introduction

Photonic integrated circuits open the way to realize compact optical phased array (OPA) devices for low-cost compact LiDARs, required for self-driving cars. Instead of using a mechanical rotating mirror, beam scanning is available by varying the phase distribution of light transmitted through the optical waveguide array^1–4^. OPA beam scanners are also useful for various applications such as free-space optical link^4^, image projection^5,6^, and lens-less imaging^7,8^.

Silicon photonic devices with a small guided mode and narrow waveguide pitch enable wide scanning of the diffracted beam from the waveguide array. In addition, increasing the arrayed waveguide channels reduces the beam divergence angle and improves the scanning resolution. A beam scanner with a divergence angle of 0.03° and a scanning angle of 45° was developed using an OPA with a 1024 waveguide array and 2 µm output pitch via a CMOS fabrication process^9^. Moreover, an aperiodic waveguide array was proposed to improve the beam scanning angle up to 90° by suppressing the side mode^10,11^. Subsequently, a high-speed phase modulator was incorporated based on charge density modulation in InAlGaAs diodes to improve the frame rate; however, the charge-induced absorption distorted the beamforming^12–14^.

Higher OPA output powers are preferred for longer detection ranges of the LiDAR. However, in silicon photonic devices, the strong confinement of waveguide mode caused nonlinear phase modulation depending on the optical power^1,14^. In addition, as the number of OPA channels increases, thermal crosstalk between the densely packed phase modulators raised a serious problem, resulting in complicated beamforming process^4,10,15^. The initial phase distribution of the output light passing through the phase modulator array was barely uniform owing to the unavoidable fluctuations of the waveguide pattern widths in the fabrication of extremely narrow silicon waveguides. Hence, a procedure of phase distribution adjustment, so-called initial beamforming, should be implemented before the scanning operation; further, the information of the calibration phase was stored in a look-up table (LUT)^1,15^. Silicon photonic devices required beamforming information for each scanning grid point, consuming the memory and limiting the number of beam scanning points capacity^14^.

Silicon nitride waveguides, owing to their small refractive index contrast, have much wider core size than the silicon waveguides and have negligible nonlinear effect due to the high optical power^16–19^. However, the thermo-optic (TO) effect of the silicon nitride is substantially low, and thus, an efficient phase modulator array cannot be produced. Hence, a hybrid device integrating silicon phase modulators was proposed^20^.

Polymeric waveguide devices have been investigated since the development of high-speed modulators with electro-optic polymers^21–23^. Recently, fluorinated polymer materials with low optical loss have been developed for producing various photonic devices useful in optical communications and optical sensors, owing to their magnificent refractive index tuning capability^24–32^. In particular, a tunable laser comprising a polymeric Bragg grating exhibited a wide wavelength tuning range and was recently adopted for WDM optical communication modules for 5G fronthaul networks^33^. Such a mature polymeric-integrated optics technology could be applied to produce OPA devices by virtue of the highly efficient TO effect and strong optical power handling capacity without the concerns of the nonlinear effect^30^. Moreover, the polymeric OPA could be integrated with the polymeric wavelength-tunable laser to produce compact solid-state beam scanners. We demonstrate two-dimensional beam scanning in this work by incorporating the polymeric wavelength-tunable laser and the polymeric OPA device, which paves the way for compact integrated polymeric beam scanner devices.

## Device design and fabrication

**Figure 1:**
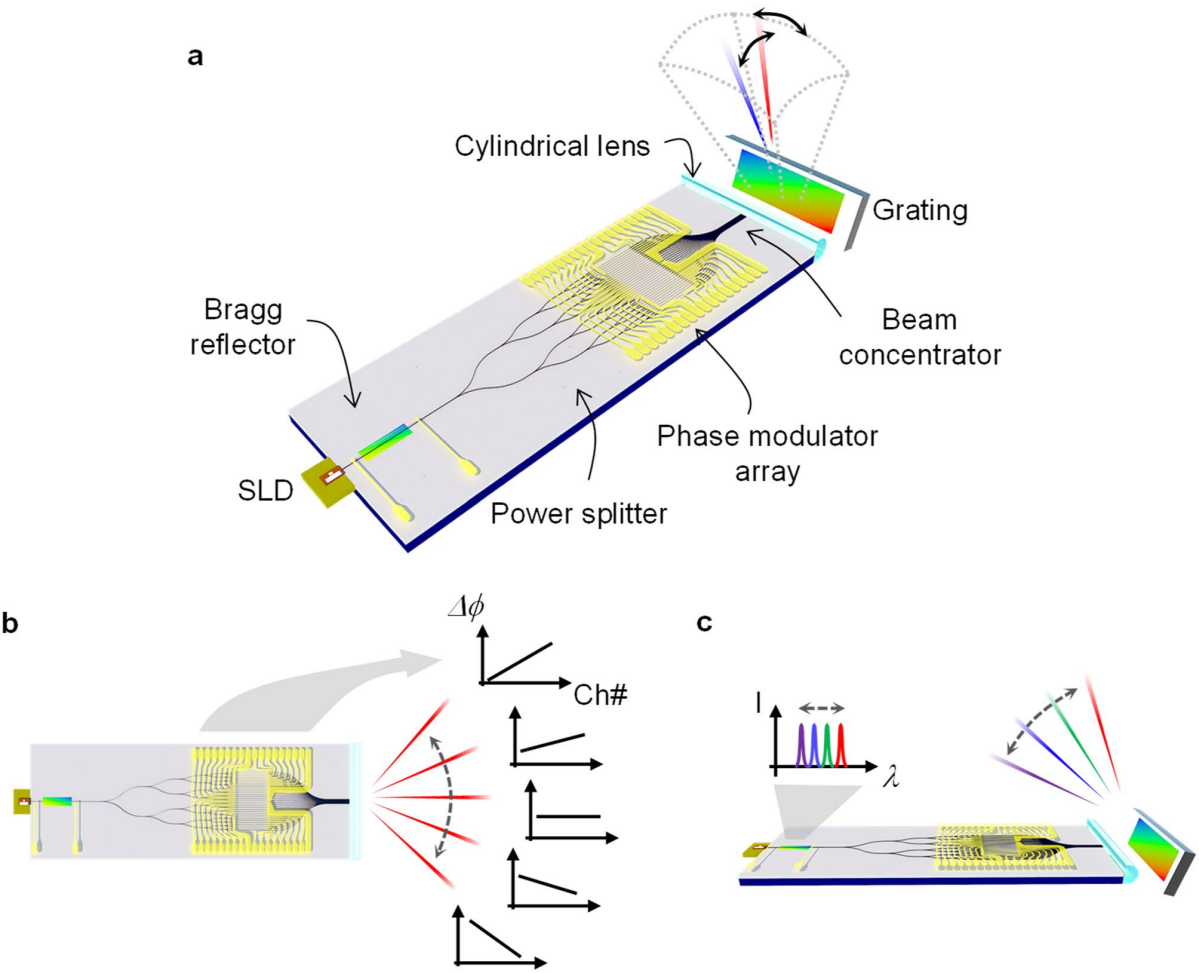
Schematics of the polymer waveguide-based OPA beam scanner with an integrated laser. (**a**) 2D beam scanner device consisting of the Bragg reflector, 1 × 32 power splitter, phase modulator array, and beam concentrator fabricated on a single chip. SLD chip, cylindrical lens, and diffraction grating are aligned and attached around the chip. (**b**) Horizontal beam scanning produced by controlling the phase distribution of the waveguide array through the polymeric phase modulator array. (**c**) Vertical beam scanning obtained by wavelength tuning of the integrated tunable laser and external diffraction grating.

A beam scanner based on the polymer waveguide consists of a tunable laser and an OPA device integrated on a single chip, as shown in Fig. 1a. The chip contains a polymer waveguide Bragg reflector used for the tunable laser, 1 × 32 power splitter, phase modulator array, and beam concentrator. Additional components of a superluminescent diode (SLD), a cylindrical lens, and a grating are attached near by the chip. The SLD and Bragg reflector consists an external cavity laser, and the lasing wavelength is tunable by the TO refractive index tuning of the polymer waveguide Bragg reflector. The light is divided into 32 channels and passes through the phase modulator array, in which the phase of guided light is controlled to produce a phase distribution for the horizontal beam scanning as depicted in Fig. 1b. The larger the number of channels, the less divergent angle of the output beam. However, increasing channel number makes it difficult to characterize the device and control the phase modulator array, which is limiting the number of channels to 32 for this first demonstration of polymeric OPA device. The polymer waveguide phase modulator has a simple structure of straight polymer waveguide with a micro-heater placed on top of the waveguide cladding. The phase modulated light is concentrated to have small pitches of array and radiated toward the external grating. Then the light is diffracted to a certain angle depending on the wavelength of the tunable laser which could produce vertical scanning as depicted in Fig. 1c.

**Figure 2:**
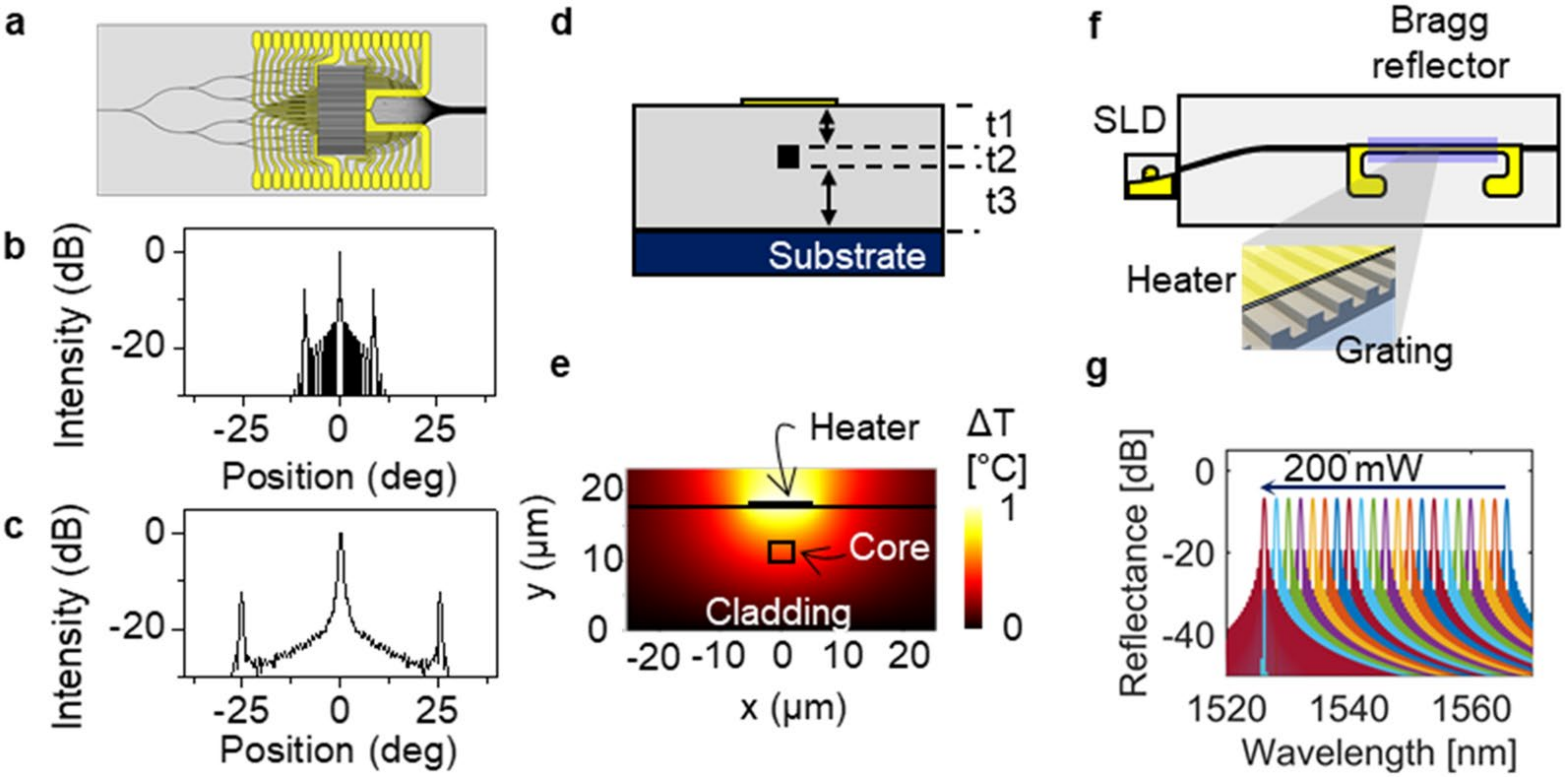
Design of the polymer waveguide beam scanner device. (**a**) Photomask layout for fabricating the 1 × 32 splitter, phase modulator array, and beam concentrator. (**b**) Far-field pattern of the 32 channel OPA device with an array pitch of 10 µm. (**c**) Far-field pattern produced by incorporating a high contrast polymer waveguide with the reduced waveguide pitch of 3.5 µm. (**d**) Cross-section of the TO phase modulator with a thin film heater located on top of the waveguide. (**e**) Heat distribution across the polymer waveguide. (**f**) External cavity tunable laser consisting of a polymeric Bragg reflector and SLD chip. (**g**) Reflection spectra of the Bragg grating tuned by the TO effect, exhibiting a tuning efficiency of 0.20 nm/mW.

Conventional polymer materials exhibit absorption loss due to the vibration overtone of the C–H bond in the wavelengths of optical communication. Fluorine-substituted polymers such as CYTOP^34,35^, LFR^27^, and fluorinated polyimide^36^ have been developed to reduce the C–H vibration absorption. Perfluorinated LFR polymers (ChemOptics, Co.) with refractive indices of 1.397 and 1.372 were incorporated for the core and cladding of the proposed polymer waveguide devices, respectively. The single-mode waveguide condition was satisfied in both TE- and TM-modes for the core size of 3 × 3 µm^2^. As an initial version to investigate the feasibility of a polymer OPA device, the number of channels in the array element was designed to be 32. The device operates for TE polarization, which is effective for the external grating. The Y-branch splitter has negligible loss compared to the propagation and coupling losses. The photomask layout of the OPA was prepared as shown in Fig. 2a. The pitch of the output waveguide array should be decreased to increase the scanning range; however, a narrow pitch caused crosstalk between waveguides. Beam propagation method (BPM) simulation was conducted to determine the optimum pitch, which was found to be 10 µm, ensuring − 10 dB crosstalk after 1-mm propagation. Consequently, the side lobe appeared at 8.9°, limiting the scanning range (Fig. 2b). However, if a high refractive index polymers are available for the core polymer in the next experimental step, one can reduce the pitch to 3.5 µm (see “Methods” for details), moving the side lobe to 25° (Fig. 2c). The TO phase modulator consists of a heater of a thin Au-Cr metal film placed on top of a polymer waveguide, as shown in Fig. 2d. The temperature gradient across the waveguide core was produced by Joule heating, as shown in Fig. 2e, and the TO effect produced corresponding refractive index distribution. Owing to the excellent TO effect and thermal confinement of polymers, the power *P*_*π*_ for π-phase change was designed to be as low as 2.1 mW (see “Methods” for details).

The polymeric tunable laser was designed by incorporating the same polymer material used for the OPA. The Bragg grating was located on top of the core, as shown in Fig. 2f, which produced a reflection peak in the C-band (1530–1565 nm). Fifth-order Bragg grating was adopted for this experiment, which can be fabricated via conventional photolithography instead of laser interferometry. When the grating had an etching depth of 350 nm and a length of 2.0 mm, according to the transmission matrix calculation, the reflectivity became 20% which was a sufficient amount for producing external feedback lasers. The tuning of the reflection spectrum was observed when the heating power of Bragg grating was increased in steps, as shown in Fig. 2g. The wavelength tuning efficiency was 0.20 nm/mW.

Vertical beam scanning was conducted by aligning the output beam of the wavelength-tunable laser to the blazed diffraction grating. For a blazed grating with a grating period of 1.0 µm and a blaze angle of 39°, the diffraction angle was calculated according to the k-vector diagram (See “Methods” for details). Beam scanning angle in the vertical direction up to 25° was possible when the wavelength was tunable for 100 nm. In addition, the diffraction efficiency was over 90% by virtue of the blazed grating.

Based on the design details, OPA devices and tunable lasers were fabricated using LFR polymers through standard fabrication processes such as spin coating, photolithography, and plasma etching (see “Methods” for fabrication details). Compared to semiconductor materials, polymer waveguides have a relatively low refractive index, and thus, the fifth-order Bragg grating period was approximately 2.8 µm, which could be fabricated via conventional photolithography.

## Device characterization: polymer waveguide, beamforming, and beam scanning

To evaluate insertion loss and high-power capacity of the fabricated polymer device, through a high NA fiber (UHNA3, Nufern, Co.), distributed feedback (DFB) laser light was coupled to a straight polymer waveguide. For the 2-cm long polymer waveguide, the insertion loss was measured to be 1.2 dB, which could be caused by 0.3 dB/facet coupling loss and 0.3 dB/cm propagation loss. The high-power handling capacity of polymer waveguide was evaluated using an erbium-doped fiber amplifier (EDFA) producing up to + 15.0 dBm optical power; no variation in the insertion loss of polymer waveguide was observed. Subsequently, + 30.0 dBm CW output light from a high-power EDFA was coupled to the device for 160 h, and no degradation was present (Supplementary Fig. 3).

By combining each of the two outputs of a 32-channel OPA device, a 16-channel Mach–Zehnder (MZ) interferometer device was produced on the same wafer to evaluate the fabrication uniformity of TO phase modulators. The *P*_*π*_ was measured as 1.8–2.0 mW, which was slightly lower than the design. The phase modulator did not cause additional optical loss. However, we found that the initial phase states of the MZ array were random, owing to the inevitable waveguide width fluctuation due to the fabrication errors. The initial phase differences should be equalized through the beamforming process (see “Methods” for details).

**Figure 3:**
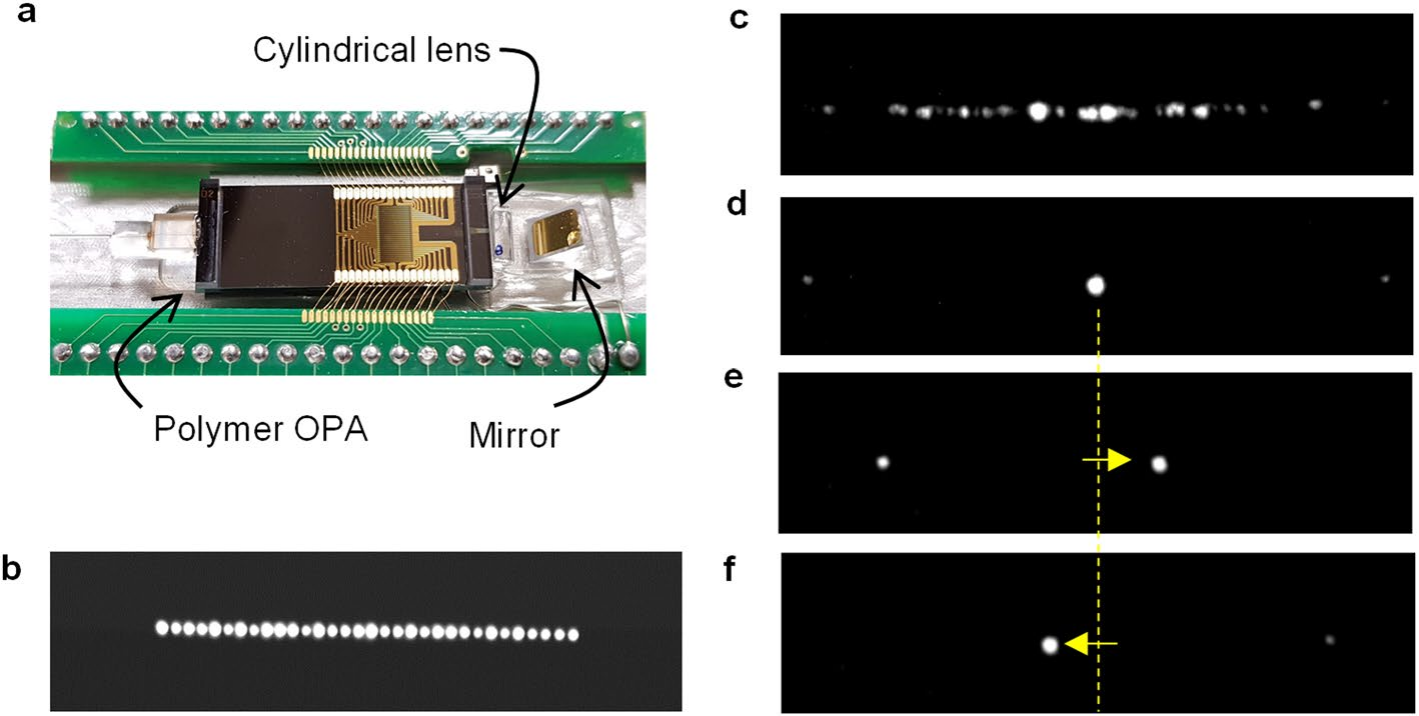
Polymer waveguide OPA characterization. (**a**) Polymer waveguide chip attached on a TEC package along with the fiber pigtail on the input, cylindrical lens for vertical collimation, and 45° mirror. (**b**) Near-field pattern of the output modes of the polymer waveguide array. (**c**) Far-field pattern scattered in the horizontal direction caused by the initial random phases. (**d**) After performing the beamforming procedure, a well-focused main beam was produced, as well as weak side lobes. (**e**, **f**) Beam scanning in the left and right directions obtained by applying a phase slope signal in addition to the beamforming phases.

The fabricated polymer chip was attached to a thermoelectric cooler package along with the pigtailed fiber on the input, cylindrical lens (Hamamatsu’s fast axis collimator lens) for vertical collimation, and 45° mirror for beam path change, as shown in Fig. 3a. The near-field pattern was observed by adjusting the objective lens close to the waveguide end-facet (Fig. 3b), while the far-field pattern was imaged after the beam was propagated 5-cm through the cylindrical lens and another imaging lens, as shown in Fig. 3c. The beam after passing the cylindrical lens becomes a collimated state only in the vertical direction and formed a far-field image at the CCD camera by passing through a 5 × objective lens and a convex lens that has a focal length of 50.2 mm. Scattered light along the horizontal line was observed initially. Following the beamforming to equalize the initial phase fluctuation, a well-focused beam was obtained, as shown in Fig. 3d. We developed a fast beamforming algorithm based on the rotating element vector (REV) method^37,38^. A bright spot among scattered far-field light was produced as a result of constructive addition of the output lights from each waveguide, whose phase was dominant compared to other scattered light. When each channel was independently phase-modulated, the influence on the dominant phase was determined by observing the intensity modulation ratio appearing at the bright spot. Then, the phase difference was compensated in a single step.

The side lobe appeared at 8.9° from the main lobe. The loss from the input power to the main beam power was measured to be − 5.2 dB (see “Methods” for details). For scanning the beam in horizontal direction, an additional phase slope was imposed on the phase modulator array, as shown in Fig. 3e, f. In these figures, the intensity of side lobe is clearly shown compared to the bright main lobe, in which no additional weak spots are appearing during the beam scanning because the beam forming condition is maintained throughout the scanning. (represented in Supplementary Movie S1 and summarized in Supplementary Fig. 5).

**Figure 4:**
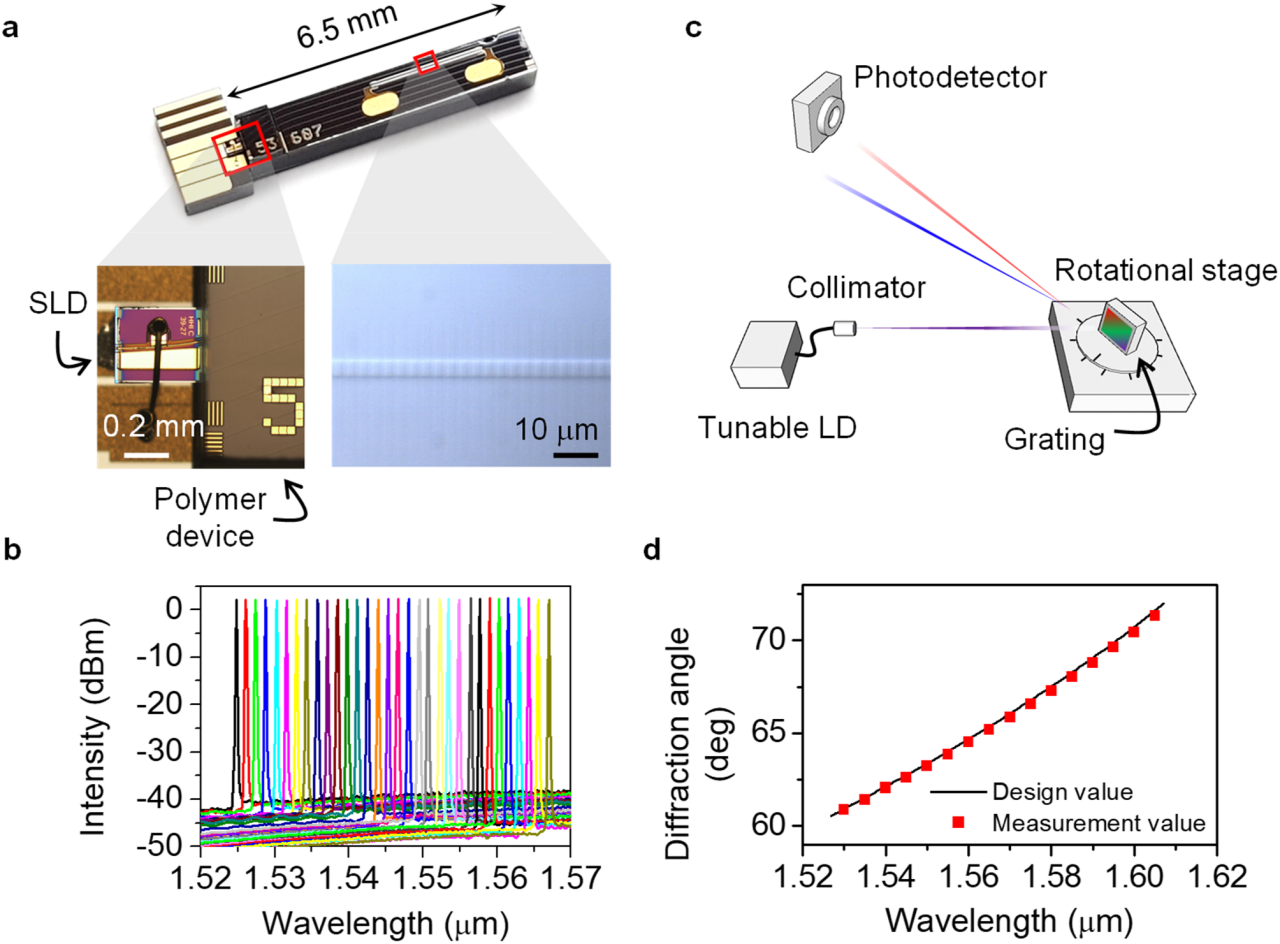
Polymer waveguide tunable laser and beam scanning through the blazed grating. (**a**) Tunable laser chip connected to the SLD chip to form an external cavity laser. (**b**) Output spectra obtained by wavelength tuning through heating the electrode of the polymer Bragg grating. (**c**) Experimental setup to measure the diffraction angle variation due to the wavelength tuning, where the laser output was incident at the Littrow angle of the blazed grating. (**d**) Diffraction angles depending on the tuned wavelength, where 14.7° scanning was obtained for 95 nm wavelength tuning.

The polymeric wavelength-tunable laser consisted of a waveguide Bragg reflector and an SLD gain chip, as shown in Fig. 4a. The output spectra of the tunable laser were measured depending on the applied current on the thin film heater, as shown in Fig. 4b. The laser output was collimated and incident on the blazed grating with a period of 1.0 µm and Littrow angle of 41° as in Fig. 4c. When the tunable laser equipment (Santec) was tuned by 95 nm, the diffraction angle changed by 14.7°, as shown in Fig. 4d. However, the polymeric tunable laser presented a wavelength tuning range of 1538–1568 nm, and the resulting scanning angle was 3.9°. Owing to the excellent diffraction efficiency of the blazed grating, the optical loss of the diffracted beam was as low as 1 dB over the entire wavelength range.

**Figure 5:**
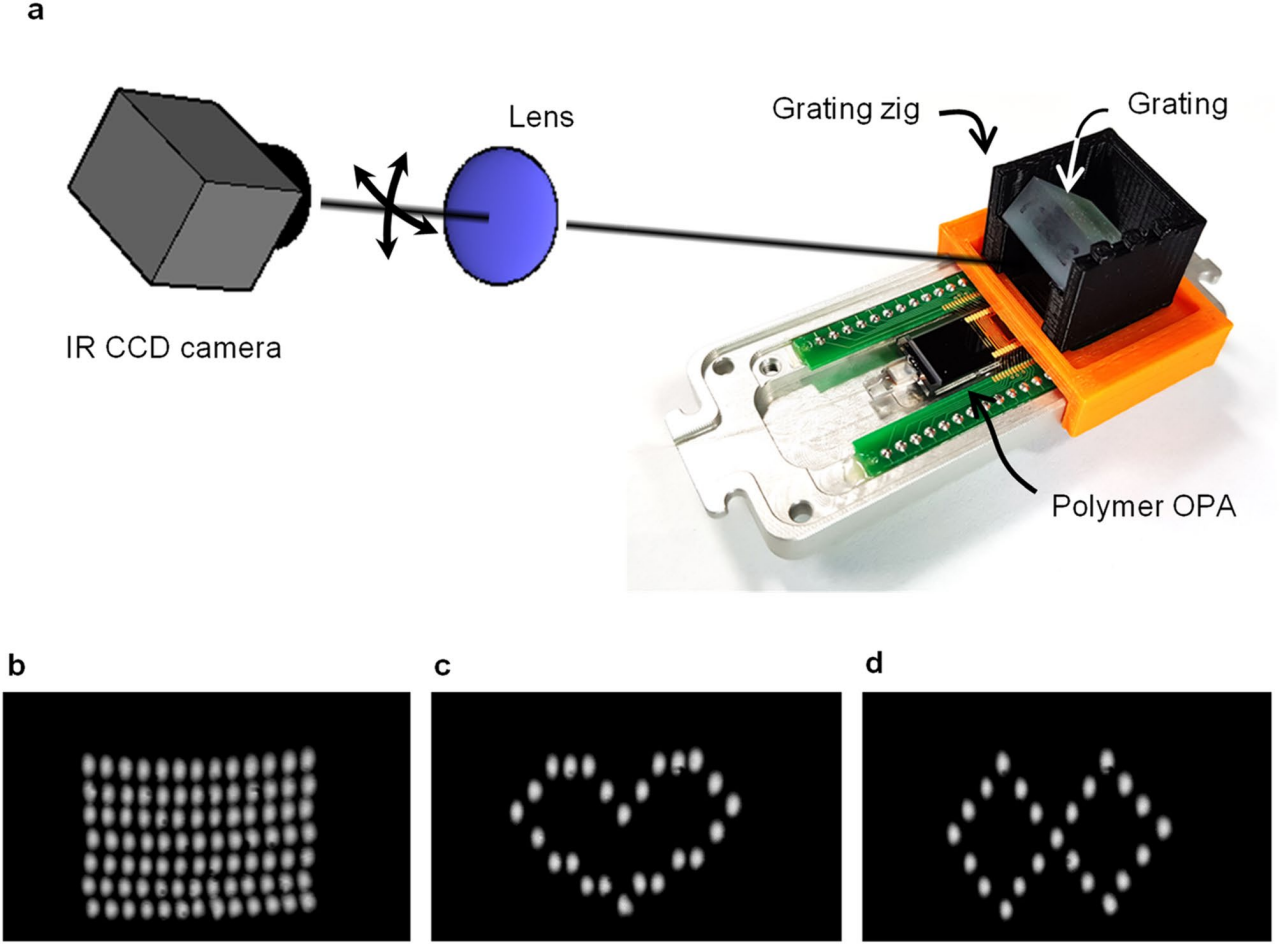
2D beam scanning experiment. (**a**) 2D beam scanner constructed using a polymer tunable laser as a light source of the polymer waveguide OPA and aligning a blazed grating over the device. Scanning light was observed by using a CCD through an objective lens. (**b**) Through the control of the wavelength and the phase distribution simultaneously, 2D scanning images were produced to match the coordinates of 13 × 7 points. (**c**, **d**) Beam scanner was controlled to produce heart pattern and figure-8 pattern based on LUT prepared in the experiment of (**b**). Movie information for (**b**–**d**) is provided as supplementary information.

A two-dimensional beam scanning was developed by incorporating the polymeric tunable laser as a light source of the OPA. The blazed grating was placed over the polymer OPA, as shown in Fig. 5a. The integration of the laser and OPA in a monolithic device was remained as a future task, and this time they were connected through an optical fiber with a small core to reduce the coupling loss. The coupling loss between high contrast small core fiber and the polymer OPA waveguide is reduced to be less than 1.0 dB by adjusting the mode profiles. The output beam from the phase modulator array was aligned to the external grating at the Littrow angle by using a 3D printed jig. The scanning beam was observed by a CCD through 5 × objective lens. Beamforming was required for each wavelength of the tunable laser because of the wavelength dispersion of the polymer waveguide, and a LUT was created for each wavelength. By controlling the wavelength-tunable laser and the phase modulator array simultaneously, 13 × 7 grid points shown in Fig. 5b were obtained. Moreover, a heart pattern (Fig. 5c) and figure-of-eight pattern (Fig. 5d) were produced (See “Methods” for details). The real-time beam scanning is also presented in Supplementary Movies S2–S4.

The response time of the beam scanning was measured by controlling the phase distribution and the wavelength, respectively. First, the beam was aligned to a fast response photodetector. Subsequently, the scanner was operated in the vertical and horizontal directions sequentially to produce an optical power modulation. Scanning response time in the horizontal and vertical directions was 7 and 21 ms, respectively (see “Methods” for details). The response time of the TO phase modulator could be improved by reducing the waveguide dimension, incorporating a high refractive index polymer.

## Conclusion and outlook

This paper proposed an OPA device that can be integrated with a tunable laser based on highly fluorinated polymer materials. Two-dimensional beam scanning was realized via phase distribution control and wavelength tuning. Fluorinated polymer waveguides could handle strong optical power with low insertion loss and were suitable for developing high-power OPA-based LiDAR. By using an external grating, the diffraction efficiency was increased, optical loss was minimized, and scanning range depending on wavelength tuning was improved. Owing to the large TO effect and low thermal crosstalk of the polymeric phase modulators, the initial beamforming was sustained during the beam scanning, exhibiting a significant advantage over silicon OPAs. The response time of the polymeric TO modulator should be improved to tens of µs by introducing high contrast waveguide to improve the frame rate^39–42^. In the next step, the tunable laser and OPA will be integrated on a single chip to realize a truly compact OPA beam scanner. Moreover, the scanning angle will be extended by incorporating the high contrast polymer waveguide. Finally, the visible wavelength transparency of the polymer will be appreciated in applications such as depth measurement LiDAR, neural probes, and optical tweezers that require visible light^5,6,43–46^.

## Methods

### Waveguide crosstalk and scanning range calculation

A beam propagation method simulation (Optodesigner, Rsoft) was conducted to determine the waveguide pitch in the beam concentrator of the OPA device. Input light was passed into one waveguide between two adjacent straight waveguides; thus, the intensity of the light coupled to the other waveguide was measured after 1 mm of propagation (see Supplementary Fig. 1). When refractive indices of 1.397 and 1.372 were used for the core and cladding, respectively, the crosstalk was calculated to be − 10 dB for a waveguide distance of 10 µm. To further reduce the waveguide pitch, one can employ high refractive index polymers containing inorganic nanoparticles such as ZrO_2_ and TiO_2_^47^, and sulfur-based polymers^48^. If the core index were increased to 1.8, the single-mode waveguide could be reduced to 0.8 × 0.6 µm^2^, and the array pitch could be reduced to 3.5 µm, preserving the low crosstalk.

### Phase modulation efficiency

To calculate the power efficiency of the polymeric phase modulator, heat distribution was calculated using the finite element method (Optodesigner Software). Moreover, *P*_*π*_ was calculated by adopting the TO coefficient of the polymer. The thicknesses of the polymers under the heater constituting the waveguide represented as t1, t2, and t3 in Fig. 2d were set to 9, 3, and 6 µm, respectively. Thermal conductivity of 0.2 W/mK and TO coefficient of − 2.5 × 10^−4^/°C were used in the calculation^27^, and heater width was 10 µm. The detailed theorical and experimental studies of TO phase modulator are described in references^25,27,30^.

### Diffraction angle of blazed grating depending on the wavelength tuning

For the incidence angle *θ*_*i*_ shown in the *k*-vector diagram of Supplementary Fig. 2a, the diffraction occurred in *θ*_*d*_, while the reflection was suppressed, owing to the blazed grating. For the light wavelength *λ*, and the grating period $\Lambda$, the diffraction angle is given by

1 $$ \theta_{d} = \sin^{ - 1} \left( {\frac{{k\sin \theta_{i} - K_{g} }}{k}} \right) $$

where *k* = 2*π*/*λ* and *K*_*g*_ = 2*π*/$\Lambda$. For the input wavelength range of 1530–1630 nm, the result of diffraction angle variation (Δ*θ*_*d*_) according to grating periods and input angles is shown in Supplementary Fig. 2b. For the blaze angle of 39° and the period of 1 µm, the diffraction angle variation of 25° was obtained for the input wavelength tuning range of 100 nm.

### Polymer waveguide OPA fabrication

Polymer waveguide fabrication started from spin coating a lower cladding polymer material onto a silicon substrate. Subsequently, it was cured by UV and backed at 160 °C for 0.5 h. Then, the waveguide pattern was formed through a photolithography process, and the pattern was transferred into the lower cladding through the oxygen plasma etching to form a groove pattern. The core polymer was coated to fill up the groove, and the entire surface was etched to leave the core in the form of a channel. The waveguide structure was completed by spin coating and curing the cladding polymer over the core; the heater pattern was formed by depositing and patterning Cr-Au with 10–100 nm thickness on the cladding layer. In the fabrication of polymeric wavelength-tunable lasers, the single-mode waveguide dimension was the same as that of the OPA device. The only difference was the grating pattern placed on the core produced by photolithography and oxygen plasma etching.

### Characterization of TO phase modulators

For the purpose of TO phase modulator characterization, a 16-channel Mach–Zehnder (MZ) interferometer array was fabricated along with the OPA by combining each of the two outputs of 32-channel OPA outputs (Supplementary Fig. 4a, b). The resistance of the fabricated phase modulator was within the range of 46–53 Ω. Each phase modulator was operated to produce the output interference signal from the MZs (Supplementary Fig. 4c). The initial phase states of each phase modulator were obtained by comparing the measured output waveforms (Supplementary Fig. 4d). The initial phase states were random due to the inevitable fluctuation of the waveguide width during the fabrication.

### Insertion loss of polymer waveguide OPA

To measure the overall optical loss of the polymer OPA beam scanner, a tunable laser equipment (Santec) was used. The powers of the main beam and side lobes were measured at a distance of 6 cm after the beamforming procedure. The main beam exhibited a loss of 5.2 dB, and the side lobes exhibited a loss of 9.8 dB; thus, the total throughput loss became 2.8 dB. The insertion loss of the polymer OPA chip was measured to be 2.0 dB considering a loss of 0.8 dB due to the collimation lens and mirror. In a straight polymer waveguide, the insertion loss was 1.2 dB, from which the additional loss was estimated as 0.8 dB for the 1 × 32 power splitter and the beam concentrator.

### Two dimensional beam scanning measurement method

The scanning beam was monitored using a CCD camera and 5 × objective lens. For each wavelength, beamforming was conducted to obtain the initial phase information and stored as an LUT. During the vertical scan performed by tuning the laser wavelength, the beamforming phase information was obtained from the LUT to compensate for the phase distortion caused by the wavelength dispersion of the polymer waveguide. The vertical scanning range was 6.0°, limited by the wavelength tuning range, 30 nm, of the polymeric tunable laser. By adjusting the incidence angle on the blazed grating from 41° to 36.6° of blazed angle, the scanning angle was improved from 3.9° to 6.0°. When the wavelength was tuned by 5 nm in each step, the scanning angle was tuned by 1.0°.

Horizontal beam scanning was conducted by adding a sloped phase distribution to the phase of the initial beamforming. The scanning resolution was roughly determined by the size of the spot image captured by the CCD. When the phase slope was increased by steps of 15°, the scanning angle changed by 0.7° in the horizontal direction.

### Response time of beam scanning

To measure the scanning response time, the OPA output beam was aligned to the photodetector with an aperture size of 0.3 mm (Supplementary Fig. 6a). Subsequently, the polymer phase modulator array was modulated to scan the beam out of the detector by applying a rectangular waveform of 0.2 s. The PD signal was measured (Supplementary Fig. 6b) to determine the falling time of 7 ms. Vertical beam scanning speed was measured by operating the polymeric tunable laser in the same setup. Heating power on the Bragg reflector tuned the wavelength and moved the diffracted beam out of the detector. A rectangular waveform signal of 50 mW peak power with a period of 0.2 s was applied to the heater to produce the wavelength change of 30 nm. The detected signal exhibited a falling time of 21 ms (Supplementary Fig. 6c).

## Supplementary Information

Supplementary Information 1.

Supplementary Video 1.

Supplementary Video 2.

Supplementary Video 3.

Supplementary Video 4.
