# Supplementary material for: Compact solid-state optical phased array beam scanners based on polymeric photonic integrated circuits
Source: Sci Rep. 2021 May 19;11:10576. doi: 10.1038/s41598-021-90120-x (PMC8134440; doi:10.1038/s41598-021-90120-x)
Supplement: Supplementary file 1 — Supplementary Information 1. [file 41598_2021_90120_MOESM1_ESM.docx]

**Supplementary Information**

**Compact solid-state optical phased array beam scanners based on polymeric photonic integrated circuits**

**Sung-Moon Kim, Eun-Su Lee, Kwon-Wook Chun, Jinung Jin, and Min-Cheol Oh***

Department of Electronics Engineering, Pusan National University, Pusan (Busan) 46241, Republic of Korea

*Corresponding author: mincheoloh@pusan.ac.kr

**Supplementary figures**

**Supplementary Figure 1 |** **BPM simulation results to calculate optical power crosstalk according to the pitch of the waveguide array.** In two adjacent straight optical waveguides, input light was launched on a waveguide; thus, the intensity of the light coupled to another waveguide was measured after 1 mm propagation. As a function of waveguide pitch, the results are shown for two cases of different refractive index contrast between the core and the cladding polymer materials. In the case of high contrast waveguide, even if the array pitch was reduced to 3.5 μm, the crosstalk was maintained less than -10 dB.

**Supplementary Figure 2 |** **Diffraction angle of the blazed grating according to the wavelength tuning.** **a**, k-vector diagram to explain the diffraction angle variation (*Δθ_d_*) of the grating according to the wavelength change from *λ_1_* to *λ_2_*. **b**, For the input wavelength range from 1530 nm (*λ_1_*) to 1630 nm (*λ_2_*), the diffraction angle variation (*Δθ_d_*) was calculated according to the grating periods (*Λ*) and the input angles (*θ_ι_*). For the blaze angle of 39° and the period of 1 μm, the diffraction angle scanning range was calculated to be 25°.

**Supplementary Figure 3 | High power handling capacity evaluation.** **a**, Output power of a straight polymer waveguide was measured while increasing the input power up to +15 dBm, using an EDFA light source. The output power was linearly proportional to the input power, and the insertion loss did not change. **b**, 1-W optical power output from a high power EDFA light source was launched on three polymer devices for 160 h; the insertion loss change was negligible.

**Supplementary Figure 4 | TO phase modulator characterization using a 16 channel MZ array. a,** CAD drawing for fabricating a 16-channel MZ device developed by connecting each two outputs of the 32 channel OPA. **b**, Photograph of the packaged device. **c**, Optical interference signal measured from the 16 MZs by applying the modulation signal on each of the 32 phase modulators. **d**, Initial phase state of each phase modulator obtained from the result of (c).

**Supplementary Figure 5 | Phase distribution control signal for the horizontal beam scanning.** **a**, Phase slope signals applied to the phase modulator array for the beam scanning after beamforming for 3 different phase slopes. **b**, Captured CCD images from the movie of the scanning beam appearing in proportion to the phase slopes. **c**, Intensity profile along the horizontal direction obtained from the images of (b). It was confirmed that during the scanning operation the beam forming was maintained clearly and simple method to impose phase slope was successful for the beam scanning.

**Supplementary Figure 6 | Response time of the polymeric beam scanner. a**, Measurement setup to find the horizontal and vertical beam scanning speed. **b**, PD output signal during the horizontal beam scanning by applying a square wave signal with a period of 0.2 s; the rise time was 7 ms. **c,** PD signal obtained by the vertical beam scanning with a signal equal to that applied in (b); the rising time was 20 ms.
